# Pregnancy-associated exposure reduction to medications used for antiphospholipid syndrome: a systematic review

**DOI:** 10.1016/j.ero.2025.03.001

**Published:** 2025-04-16

**Authors:** Judith Zarek, Avery Brydon, Karen Spitzer, Carl Laskin, Shinya Ito

**Affiliations:** 1 Translational Medicine, The Hospital for Sick Children Research Institute, Toronto, ON, Canada; 2 Department of Pharmaceutical Sciences, University of Toronto, Toronto, ON, Canada; 3 Applied Clinical Pharmacology, Department of Pharmacology and Toxicology, University of Toronto, Toronto, ON, Canada; 4 TRIO Fertility, Toronto, ON, Canada; 5 Division of Rheumatology, Department of Medicine, University of Toronto, Toronto, ON, Canada; 6 Department of Obstetrics and Gynecology, University of Toronto, Toronto, ON, Canada; 7 Department of Pharmacology of Toxicology, University of Toronto, Toronto, ON, Canada; 8 Division of Clinical Pharmacology and Toxicology, Department of Paediatrics, The Hospital for Sick Children, Toronto, ON, Canada

## Abstract

**Objectives:**

Pregnancy-associated hypercoagulability and potential changes in drug disposition increase the risk of anticoagulant treatment failure for pregnant women with antiphospholipid syndrome (APS), which is characterised by thrombotic and obstetric complications. We conducted a systematic review of pregnancy-associated pharmacokinetic (PK) changes of medications commonly used for APS.

**Methods:**

We systematically searched MEDLINE, Embase, Cochrane Central Register of Controlled Trials, Web of Science, and Pharmaceutical Abstracts. Publications of PK assessment in pregnant individuals compared to nonpregnant controls were included. Six target drugs in 3 groups were low-dose aspirin, heparins (unfractionated heparin and low molecular weight heparin), and antimalarial (hydroxychloroquine and chloroquine). PK parameters in pregnancy including apparent clearance (CL) were compared to those of the nonpregnant state.

**Results:**

In 3460 screened publications, we identified 12 articles. Of the 3 drug groups, only heparins included studies of pregnant women with APS. Despite the diverse PK profiles of these drugs, all 6 drugs showed statistically significant changes of 1.5- to 2-fold represented by decreased area under the curve, lower peak concentration, and/or increased CL with large interindividual variations. Details of postpartum recovery profiles of the PK changes were not reported in any of the studies.

**Conclusions:**

Limited evidence shows the reduction of exposure to APS medications during pregnancy, implying a higher risk of therapeutic failure. Although the clinical impact of the PK changes awaits sufficiently powered studies, the lack of PK data in pregnancy and postpartum remains a significant challenge in women's health.

**WHAT IS ALREADY KNOWN ON THIS TOPIC**

• Current guidelines for the treatment of antiphospholipid syndrome (APS) during pregnancy consider the use of heparins, antimalarials, and low-dose aspirin. Medications during pregnancy are susceptible to pharmacokinetic (PK) changes, which can influence optimal therapeutic doses. Although pregnancy-associated increase in clearance (CL) of some of the medications for APS has been suggested, detailed and systematic analyses of the evidence are lacking.

**WHAT THIS STUDY ADDS**

• Despite diverse elimination pathways, all these medications for APS show a significant decrease in exposures during pregnancy, due to increased CL by about 1.5-fold on average. However, individual variations are large, and time course of these changes antepartum and recovery profiles postpartum remain speculative.

**HOW THIS STUDY MIGHT AFFECT RESEARCH, PRACTICE OR POLICY**

• PK changes of the medications for pregnant patients with APS await further studies with a clear focus on its clinical impact. Although an average 1.5- to 2-fold increase of heparin dosing rates is needed to compensate for the reduction of their blood levels in pregnancy, large individual variations in the PK changes and risk factor profiles necessitate regular monitoring towards individualised target levels. While decreased drug exposure by a similar degree is reported for low-dose aspirin and antimalarials, whether a dose increment is necessary or not remains unknown.

Alt-text: Unlabelled box

## INTRODUCTION

Antiphospholipid syndrome (APS) is an acquired inflammatory autoimmune disease characterised by the presence of antiphospholipid antibodies and clinical manifestations of thrombosis and/or obstetrical complications, including recurrent pregnancy loss, preterm delivery, preeclampsia, and eclampsia [1]. Treatment of APS in pregnancy is challenging because pregnancy itself creates a state of hypercoagulability and often alters drug disposition.

During pregnancy, many physiological processes change, including hemodynamics (eg, increase in organ blood flow and blood volume) and production-elimination balances of endogenous substances (eg, decrease in relative counts of red blood cells and plasma protein concentration). Resultant alterations of drug disposition usually emerge as decreased protein binding, increase in volume of distribution (V), and changes in drug clearance (CL) in various combinations [2,3], often justifying dose modification or a choice of alternative drugs. For example, a decrease in albumin concentrations in blood leads to decreased protein binding (increased unbound fraction of drug), which may cause an increase in actual concentrations of unbound drugs. This is particularly pronounced in those with inherently high protein binding. In addition to changes in the activity of drug-metabolising enzymes, increased unbound fraction itself may increase the overall CL of drugs, depending on drug property and administration routes. Altered blood cell fractions in pregnancy show as low haematocrit (also known as physiological anaemia in pregnancy) and leucocytosis, affecting whole blood concentrations of some drugs, which makes interpretation of drug concentrations challenging. Enlarged V is also one of the factors to reduce peak concentrations of drug (C_max_). Combined impact of these changes on blood concentrations of drug at steady state are variable and specific to each drug. Of these pharmacokinetic (PK) parameter alterations, changes of CL are particularly important because area under the curve (AUC) and average blood concentrations at steady state are dependent on CL.

CL increase in pregnancy has clinical implications. For example, anti-HIV medications coadministered with cobicistat, a PK enhancer, are considered unsuited for pregnancy because of the difficulty in achieving effective blood concentrations at a steady state [4]. Similarly, lamotrigine for epilepsy requires frequent monitoring and dose increase due to substantial CL increase during pregnancy [5].

APS pharmacotherapy in pregnancy aims for thromboprophylaxis, including low-dose aspirin (LD-ASA), heparins (unfractionated heparin [UFH] and low molecular weight heparin [LMWH]), and antimalarial (hydroxychloroquine [HCQ]). Each of them has rather unique pharmacologic properties and elimination pathways. LD-ASA exerts its effects on platelets in the presystemic circulation while being eliminated by plasma cholinesterase. UFH and LMWH are mixtures of polymers with diverse sizes, eliminated mainly through the reticuloendothelial system with variable levels of renal excretion. HCQ is metabolised by hepatic cytochrome P450 enzymes, and its extensive distribution into blood cell components causes matrix dependency of drug concentrations in blood. Although individual reports exist investigating pregnancy-associated PK changes of these drugs for APS, in-depth analyses based on a systematic approach are lacking. Furthermore, the presence of various forms of inflammation in APS during pregnancy [[6], [7], [8]] raises a question if pregnancy-associated PK changes in women with APS are superimposed by systemic inflammation, a known PK modifier [9,10].

The previous systematic review by our group [2] elucidated the expansive landscape of the entire field of pregnancy-associated PK changes. In the present study, we take a focused approach to scrutinise pregnancy-associated PK changes of the specific groups of medications for APS. In-depth analyses are necessary to address the diverse PK profiles and complex pharmacologic properties of these medications.

## METHODS

### Overview

This review was conducted according to the Centre for Reviews and Dissemination guidance for undertaking reviews in health care [11] and followed PRISMA (Preferred Reporting Items for Systematic reviews and Meta-Analyses) statements. Although we initially used the PRISMA 2009 checklists, PRISMA was updated mid-review, and therefore, we utilised the updated checklist (Supplementary Table S1) [12]. Because the study screen and selection had already been conducted according to 2009 statements, the 2009 study flow [13] was used. This review is registered in PROSPERO (The International Prospective Register of Systematic Reviews) [14].

The overarching project includes a broad range of medications to treat rheumatic diseases. Focusing on APS, the present study is a systematic review confined to the following drugs: (i) LD-ASA; (ii) heparins (UFH and LMWH); and (iii) antimalarial drugs (chloroquine and HCQ). These medications were chosen based on the task force recommendations for use in pregnant patients by the American College of Rheumatology [15] and the European League Against Rheumatism [16]. Although chloroquine is not described in the professional practice guidelines for APS in pregnancy [15,16], we included it in our analyses due to pharmacologic similarities between chloroquine and HCQ. In addition, data from the rich history of chloroquine use in pregnant patients with malaria complement the PK information of HCQ in pregnant women.

Our systematic review focused on aggregated patient data, some of which included parameters derived from population PK analyses and their simulated values. In addition to these variations of PK analysis methodology, we noted diverse study designs in the selection of nonpregnant control, drug doses, and participants’ disease conditions. We, therefore, did not use a statistical approach of a meta-analysis but rather attempted to narratively summarise existing data with interpretation based on pharmacologic principles.

### Search strategy

Searches were conducted in MEDLINE (Ovid), Embase (Ovid), Cochrane Central Register of Controlled Trials (Ovid), Web of Science (Thomson Reuters), and Pharmaceutical Abstracts (Ovid) from database inception to June 2, 2020. The search was updated to include publications until April 1, 2024.

Database subject heading fields (MeSH: Medical Subject Headings) and text words were used for the following concepts: pregnancy AND PKs variables AND specific medications (list is available in the Supplementary Table S2). Truncation symbols were used with the text words, when appropriate, to capture variations in spelling and word endings. Subsequently, we reviewed the identified studies and examined their references to find other potential articles. Information available from relevant conferences was also reviewed. No publication date, language, or location restrictions were applied. The full search strategy for Embase can be found in Supplementary Table S3.

### Study selection

Inclusion criteria according to PICOS (Population, Intervention, Comparison, Outcomes and Study) [11] were set as described in the following.

The study has reported the following: (i) an original set of PK parameters in pregnant women exposed to an antirheumatic medication and (ii) a comparison of the PK variables between pregnant and nonpregnant individuals or states. The review did not cover animal studies or studies without original drug concentration measurements.

### Screening and article retrieval

Full texts of eligible titles and abstracts were read by 2 reviewers (JZ and AB) and judged against the inclusion criteria. If necessary, it was reviewed by a third reviewer. Two authors (JZ and AB) independently extracted data on population, intervention, and outcome variables. Disagreements were resolved by discussion, and a third author (SI) was consulted for arbitration.

### Reporting quality

The ClinPK list [17] describes a minimum set of 24 essential items for reporting PK study results and is used for the evaluation of those studies [2,18,19]. The ClinPK list was used to assess the reporting characteristics of each study after removing items 20 and 21, which are not applicable to our analyses; Item 12 was also removed if the study did not use the population PK method. For each study, the number of compliant items was expressed as a percentage of the total relevant item number.

### PK data extraction

The main PK parameter of interest was systemic CL. Other parameters, such as volume of distribution (V), elimination half-life or mean residence time (a ratio between V and CL, proportional to elimination half-life), and dose-dependent concentrations including AUC and C_max_, were also extracted. Although C_max_ may not represent a true peak level, depending on the sampling timing, we analysed it because it is sometimes used in clinical settings (eg, heparins). Because the APS medications are mostly given as oral or subcutaneous injection, CL and V in this analysis are apparent parameters, which are relative to respective bioavailability (F): CL/F and V/F. If the original mean/median values were presented in a graph format only, we extracted them using a web-based programme PlotDigitizer version 3 (https://plotdigitizer.com).

### Data analyses

Reported mean or median values of the PK variables in pregnancy were expressed as the percentage of those in the nonpregnancy control and designated as % pregnancy/nonpregnancy (%P/NP). A reported single mean summarising those at different gestational periods was taken as a representative value for pregnancy in that study. If there was a statistically significant difference between nonpregnancy and pregnancy in 1 or more comparisons in a study, the mean parameter value shown in table formats reflected statistical significance.

In most studies, nonpregnancy comparators were either nonpregnant women or nonpregnant states of the same women in a crossover manner. If multiple comparators were described in a study, we chose nonpregnant women or a crossover control of preconception states, unless otherwise stated, to avoid the transitional nature of postpartum periods.

Some studies used dose escalation during pregnancy or weight-based dosing that is a de facto dose increment due to pregnancy-related physiological weight increase. If doses were increased in pregnant groups without reporting dose-adjusted concentration parameters (AUC, C_max_, and C_trough_), we also dose-standardised them before %P/NP estimation and showed both dose-unadjusted and -adjusted values. In the case of weight-based dosing, we converted them to group-average doses using the mean body weight of each group before dose standardisation.

Apparent CL is a reciprocal of dose-standardised AUC at a steady state, and so are their derived %P/NP values. When only 1 of the 2 PK parameters was reported, we converted it to the other by taking the reciprocal.

## RESULTS

### Overall study characteristics

#### Study selection

**Figure 1:**
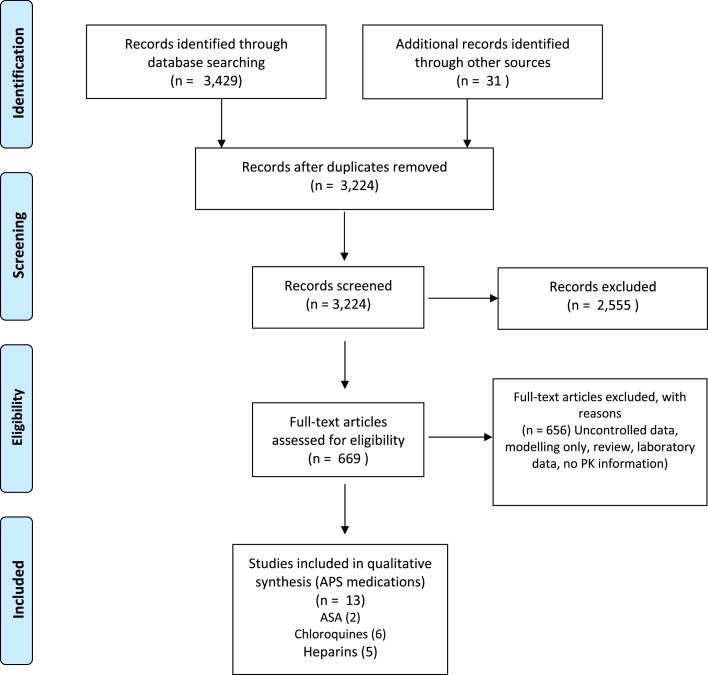
PRISMA (Preferred Reporting Items for Systematic reviews and Meta-Analyses) inclusion flow diagram. Adapted from Moher et al [13]. APS, antiphospholipid syndrome; ASA, aspirin; PK, pharmacokinetic.

The results of the literature selection are shown in the PRISMA flow diagram (Fig 1) [13]. We retrieved 3429 publications from our database search for all antirheumatic medications. An additional 31 publications were identified in the grey literature search for review. After removing duplications, 3224 articles remained to be screened. From the screening of titles and abstracts, 2555 were removed because they did not meet the inclusion criteria of this study. After assessing 669 articles against the eligibility criteria, 13 papers were identified for these medications to treat APS.

There are 2 overlapping studies of chloroquine PK [20,21]. The study by Karunajeewa et al [20] is based on the PK of chloroquine coadministered with sulphadoxine–pyrimethamine, which was later reanalysed with an additional dataset of chloroquine–azithromycin combination [21]. Because the latter study [21] reported a significant change in the apparent CL of a chloroquine metabolite in the azithromycin coadministration arm, we chose the former study by Karunajeewa et al [20] to avoid a potential impact of the interaction.

The final analyses are based on a total of 12 publications on the 6 drugs (enoxaparin and dalteparin were only LMWH identified in this analysis) and 2 of their metabolites (Table 1, Table 2, Table 3) [20,[22], [23], [24], [25], [26], [27], [28], [29], [30], [31], [32]].

**Table 1:** Low-dose aspirin

	Aspirin	Salicylic acid (aspirin metabolite)	
Author [reference #]	Rymark et al [22]		Shanmugalingam et al [23]
ClinPK compliance (%)	18/21 (86%)		14/21 (67%)
Study period in gestational weeks: wk	27-29 wk and 36-38 wk		25-26 wk
Pregnant women: N	20 healthy: 10 in each of the 2 study periods.		12 with indications for low-dose aspirin: 3 in each of the 4 groups of different doses or formulations.
Nonpregnant control: N	11 healthy volunteers (5 women and 6 men)		3 healthy women for all 4 dose groups

**PK parameters in pregnancy (% Pregnancy/nonpregnancy: %P/NP)**			

Dose and administration	Oral 75 mg single dose for PK study		Oral single dose: 100 mg or 150 mg of enteric-coated or not.
PK analyses method	Noncompartmental		Compartmental
Apparent CL: CL/F	135^a^	143^a^	**206**
T_max_	**163**	119	100
AUC	74	**70**	**63**
C_max_	**69**	**69**	**74**

AUC, area under the curve; CL, systemic clearance; CL/F, apparent oral clearance; C_max_, maximum concentration; F, bioavailability; T_max_, time to maximum concentration.

Pregnancy PK parameters are expressed as % of nonpregnancy values (%P/NP). If multiple values of a single parameter were described per dosing regimens in the original report, a mean is shown. Bold font %P/NP value indicates that a statistically significant difference between pregnancy and nonpregnancy values was described for at least one parameter value in the original reports. Otherwise, %P/NP shows values of statistical nonsignificance or those with no reported statistical analysis in original reports.

a Apparent CL was estimated from mean AUC as dose/AUC before %P/NP was derived.

#### Study subjects

Of the 3 drug groups, only heparins included studies on women with APS as study participants, but no diagnostic and history detail were provided. Two studies (LD-ASA [22] and UFH [24]) used volunteers without a disease condition. All 3 studies on chloroquine [20,30,31] were done in those with malaria or those living in a malaria endemic area.

#### PK analysis methods

Three (25%) [20,27,32] of the 12 studies used a population PK method with a compartmental structural model. Seven studies (58%) [22,[24], [25], [26],[29], [30], [31]] used model-independent noncompartmental approach, and 1 study (8%) [23] used a compartmental model-based analysis. The remaining 1 study (8%) [28] used a mixed-effect repeated measure model based on single sampling around the anticipated time point of a peak concentration.

#### Reporting quality

Conformity with the PK reporting guidelines (ClinPK checklist [17]) was greater than 80% of the relevant items in 7 of the 12 studies (58%). A median conformity rate was 84% of the checklist items (95% CI: 67%-86%).

#### Reported parameters

In 7 of the 12 publications (58%) [20,23,[25], [26], [27],^30^,^32^], apparent CL was reported for 4 drugs and 2 metabolites. Measured AUC values were described for 5 drugs and 2 metabolites in 9 publications (75%) [20,[22], [23], [24], [25], [26],[29], [30], [31]]. Similarly, measured values of C_max_ of these 5 drugs and 2 metabolites were reported in 8 publications (67%) [[22], [23], [24], [25], [26],[29], [30], [31]]. Apparent V was reported for 3 drugs and 1 metabolite in 4 of the 12 studies (33%) [20,26,27,32]. Three studies of population PK analyses [20,27,32] reported model-based simulated concentration values.

### Dose escalation and parameter standardisation

Dose escalation approaches or weight-based dosing were reported in 5 studies for 4 drugs (UFH [24,25], enoxaparin [27,28], dalteparin [25], and chloroquine [30]) and a metabolite of chloroquine (desethylchloroquine [DECQ] [30]), causing increased dosing rates during pregnancy. Of these, a study of enoxaparin by Aleidan et al [28] reported a dose-adjusted model estimate of C_max_. Because the remaining 4 studies did not report dose-adjusted concentration parameters for pregnancy, we estimated %P/NP using dose-standardised values of AUC, C_max_, and C_trough_. Both unadjusted and adjusted %P/NP values are shown in Table 2, Table 3.

**Table 2:** Heparins

	Unfractionated heparin		Low molecular weight heparin				
			Enoxaparin			Dalteparin	
Author [reference #]	Brancazio et al [24]	Ensom and Stephenson [25]	Casele et al [26]	Lebaudy et al [27]	Aleidan et al [28]	Sephton et al [29]	Ensom and Stephenson [25]
ClinPK compliance (%)	18/21 (86%)	17/21 (81%)	18/21 (86%)	19/22 (86%)	15/21 (71%)	16/21 (76%)	17/21 (81%)
Study period ingestational weeks: wk	24-30 wk	9, 22, and 34 wk	12-15 wk and 30-33 wk	Throughout	Not specified	12, 24 and 36 wk	9, 22, and 34 wk
Pregnant women: N	6 healthy	6 with APS	13 including APS	75 with thrombotic conditions including 39 APS	36 with thrombotic conditions	24 with APS	9 with APS
Nonpregnant women: N	6 healthy	6 prepregnant (crossover)	13 postpartum (crossover)	38 with DVT	32 with thrombotic conditions	16 postpartum (crossover)	9 prepregnant (crossover)

**PK parameters in pregnancy (% Pregnancy/nonpregnancy: %P/NP)**							

Subcutaneous injection dose(T: trimester)	single dose: 143 U/kg	5000 (prepregnancy); 5000-7500-10000 U/12 h (T1-T2-T3)	40 mg/d	40-60 mg/d, or1 mg/kg/12 h if high-risk	1 mg/kg/12 h	50 mg/d	25 mg/d (prepregnancy); 25-50-75 mg/d (T1-T2-T3)
PK analysis method	Noncompartmental	Noncompartmental	Noncompartmental	Population PK model (compartmental)	A mixed-effect repeated measure model for C_max_	Noncompartmental	Noncompartmental
Apparent CL: CL/F	204^a^	166	**132**	**148**		137^a^	211
Apparent V: V/F			**124**	**149**			
Mean residence time			97				
T_max_	**51**	105	108				
AUC	**55,** 49^b^	193, 126^b^	**78**	68^c^		73	**116,** 58^b^
Css			**66**				
C_max_	**48,** 43^b^	**183,** 122^b^	**76**	Simulation 69 (40 mg/d) 76 (1 mg/kg × 2/d), 68^b^	**82**^d^	**61**	**115,** 61^b^
C_trough_		200, 150^b^					217, 117^b^

APS, antiphospholipid syndrome; AUC, area under the curve; CL, systemic clearance; CL/F, apparent oral clearance; C_max_, maximum concentration; C_ss_, steady-state concentration; C_trough_, trough concentration; DVT, deep vein thrombosis; F, subcutaneous injection bioavailability; T_max_, time to maximum concentration; V, volume of distribution at steady state or in a 1-compartment model; V/F, apparent V.

Pregnancy PK parameters are expressed as % of nonpregnancy values (%P/NP). If multiple values of a single parameter were described per dosing regimens in the original report, a mean is shown. Bold font %P/NP value indicates that a statistically significant difference between pregnancy and nonpregnancy values was reported for at least one parameter value in the original reports. Otherwise, %P/NP shows values of statistical nonsignificance or those with no reported statistical analysis in original reports. Blank cells indicate no reported value.

a Apparent CL was estimated as Dose/AUC before %P/NP was derived.

b Reported values were dose-standardised as doses were increased in pregnancy.

c AUC was estimated as Dose/(apparent CL) before %P/NP was derived, thereby dose-standardised.

d This model estimate was dose-adjusted in the original report.

**Table 3:** Antimalarials

	Hydroxychloroquine	Chloroquine			Desethylchloroquine		
Author [reference #]	Balevic et al [31]	Chukwuani et al [32]	Karunajeewa et al [20]	Lee et al [30]	Chukwuani et al [32]	Karunajeewa et al [20]	Lee et al [30]
ClinPK compliance (%)	19/22 (86%)	14/21 (67%)	19/22 (86%)	14/21 (67%)	See chloroquine	See chloroquine	See chloroquine
Study period in gestational weeks: wk	Throughout	24-26 wk	median 22 wk [IQR: 20, 28]	20-32 wk			
Pregnant women: N	22 with SLE	5	30	12 with malaria			
Nonpregnant women: N	14 with SLE at postpartum	5	30	13 with malaria			
**PK parameters in pregnancy (% Pregnancy/nonpregnancy: %P/NP)**							

Dose and administration	Mostly 400 mg/d orally throughout pregnancy and postpartum	Oral 600 mg single dose	Oral 450 mg/d for 3 days plus a single dose of S-P	10-10-5 mg/kg/d orally over 3 days	See chloroquine	See chloroquine	See chloroquine
Sample matrix	Serum	Plasma	Plasma	Whole blood			
PK analysis method	Population PK model (compartmental)	Noncompartmental	Population PK model (compartmental)	Noncompartmental			
Apparent CL: CL/F	**145**	137^a^	**134**	119	11^a^	**134**	108^a^
Apparent V: V/F	100		**107**			105	
Elimination half-life	69		**90**	86		**85**	
T_max_		79		150	**357**		
AUC	70^b^	73	**75**	91, 84^c^	**932**	**55**	101, 93^C^
C_max_	Simulation: 77	75	Simulation: 79	137, 127^C^	**1194**	Simulation: 75	

AUC, area under the curve; CL/F, apparent oral clearance; CL, systemic clearance; C_max_, maximum concentration; F, oral bioavailability; SLE, systemic lupus erythematosus; S-P, sulphadoxine–pyrimethamine; T_max_, time to maximum concentration; V, volume of distribution at steady state or in a 1-compartment model; V/F, apparent V.

Pregnancy PK parameters are expressed as % of nonpregnancy values (%P/NP). If multiple values of a single parameter were described per dosing regimens in the original report, a mean is shown. Bold font %P/NP value indicates that a statistically significant difference between pregnancy and nonpregnancy values was reported for at least one parameter value in the original reports. Otherwise, %P/NP shows values of statistical nonsignificance or those with no reported statistical analysis in original reports. Blank cells indicate no reported value.

a Apparent CL was estimated by us from mean AUC in the original report as Dose/AUC before %P/NP was derived.

b AUC was estimated by us from a mean of apparent CL in the original report as Dose/(apparent CL) before %P/NP was derived, thereby dose-standardised.

c Dose-standardised values.

### Overview of PK parameter change during pregnancy

**Figure 2:**
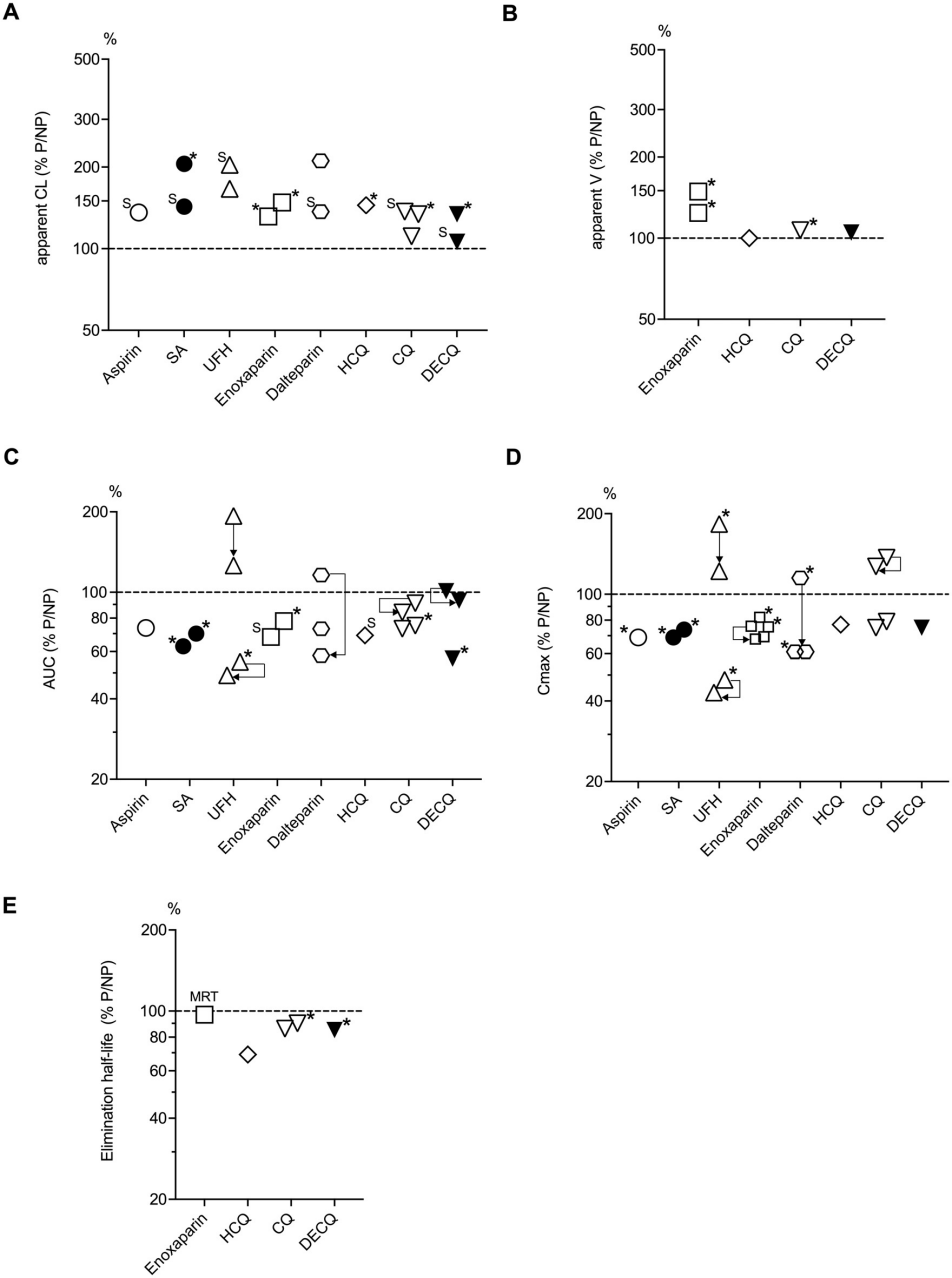
Pharmacokinetic (PK) parameter changes of medications for antiphospholipid syndrome (APS) in pregnancy. Reported mean or median PK parameter values in pregnancy are expressed as % of nonpregnant values (% pregnancy/nonpregnancy [% P/NP]). If multiple %P/NP values were described in one study, we calculated a mean. Each study value is represented by a symbol and grouped along drug categories on the X-axis (Aspirin: °; Salicylic acid (SA) as aspirin metabolite:●; Unfractionated heparin (UFH): △; Enoxaparin: □; Dalteparin: ⎔; Hydroxychloroquine (HCQ): ◇; Chloroquine (CQ): ▽; desethylchloroquine (DECQ) as CQ metabolite: ▼). Panel A: Apparent CL. Panel B: Apparent V. Panel C: AUC. Panel D: C_max_: the size of the symbol of enoxaparin is smaller to minimise the overlap of the data points. Panel E: Elimination half-life; %P/NP of mean residence time (MRT) is shown for enoxaparin. Note: Apparent clearance (CL), area under the curve (AUC) and C_max_ of DECQ reported in Lee et al [30] are outside the axis ranges and not shown. *: statistically significant increase or decrease was described in the original reports for pregnancy values compared to nonpregnant state in the original reports; ^S^: a superscript S in Panel A represents a %P/NP value estimated by us as a reciprocal of %P/NP of Dose/AUC; similarly, %P/NP with S in Panel C is estimated as a reciprocal of %P/NP of reported apparent CL. The symbols connected by an arrow show parameter values when doses were increased in pregnancy: symbols in the arrow origin represent data before dose standardization in the original reports, and those at the arrowhead are dose-standardised values calculated by us using aggregated data.

Apparent CL in pregnancy ranged from about 120% to more than 250% of nonpregnancy levels. Statistical significance of the increase was reported in salicylic acid as a metabolite of aspirin, enoxaparin, HCQ, chloroquine, and its metabolite DECQ (Tables 1-3; Fig 2A [30]). Apparent V showed a modest increase (Fig 2B). AUC and C_max_, as well as AUC/Dose and C_max_/Dose, were lower in pregnancy by 20% to 50% even when pregnancy doses were higher using body weight-based approach (Fig 2C,D). In contrast, a study for UFH and dalteparin [25] using 2- to 3-fold dose escalation based on gestational age reported statistically significant increase in C_max_ (UFH and dalteparin) and AUC (dalteparin) as expected; AUC/Dose and C_max_/Dose for UFH also remained higher than nonpregnant state while those dose-adjusted values for dalteparin were lower than nonpregnancy (Table 2; Fig 2C,D). To summarise, all 6 drugs and 2 of their metabolites showed significant changes as either increased apparent CL or decreased concentrations or both. Elimination t_1/2_ and mean residence time showed no change or modest shortening during pregnancy (Fig 2E), consistent with proportionality between half-life and a ratio of V to CL. In contrast to pregnancy-associated PK changes, none of the analysed studies addressed details of time profiles of PK parameter restoration *postpartum*.

### Drug-specific characteristics

#### Low-dose aspirin

Aspirin disposition during LD-ASA therapy is reported in 1 study of women and men without a particular disease condition [22], which showed AUC reduction in pregnancy by about 25%, although statistical significance was not reached (Table 1). C_max_ was significantly lower in pregnancy [22]. For metabolite salicylate, they showed a significant reduction of both AUC and C_max_. Similarly, the other study [23] reported a significant decrease of AUC and C_max_ of salicylic acid in pregnancy, along with a 2-fold higher apparent CL. However, clinical consequences of the exposure reduction, including platelet function, have not been fully characterised. Likewise, the time course of the changes antepartum and postpartum has not been addressed (Table 1, Table 2, Table 3).

#### Unfractionated heparin

Heparin concentrations are monitored as antifactor Xa levels, which are surrogates of prophylaxis effectiveness. There were 2 studies for UFH: 1 was a single-dose study with a body weight-based dose in healthy volunteers [24]; and the other used a 2-fold dose increase based on gestational ages in an APS treatment setting [25]. In the single-dose study [24], a significant reduction of AUC and C_max_ was observed in pregnancy by nearly half (Table 2), which was associated with significantly shorter aPTT; our estimates of AUC/Dose and C_max_/Dose were also lower in pregnancy (Fig 2C,D). Although they did not report apparent CL, our estimate implies CL increase (Fig 2A). The other study in patients with APS [25] showed significantly increased C_max_ because the dose was increased by 1.5- and 2-fold but individual variations were large, and statistical significance was not observed for changes in AUC, apparent CL, and C_trough_.[25] Our estimates using their data [24] suggest pregnancy values of AUC/Dose, C_max_/Dose, and C_trough_/Dose remain higher than nonpregnant state (Fig 2C,D; Table 2). Although this study with 2-fold dose escalation reported trimester-specific changes [25], the lack of statistical significance makes interpretation difficult.

#### LMWH

Two studies in women with APS [26,27] showed a 1.5-fold increase in apparent CL of subcutaneous enoxaparin during pregnancy, which was associated with a significant reduction of antifactor Xa AUC by about 30% (Fig 2C; Table 2). Reduction of C_max_ and C_max_/Dose was also apparent, which is a consequence of both increased CL and expanded V [26,27]. The model estimates [27] suggest further expansion of V towards the end of pregnancy consistent with even more reduction of C_max_ in the third trimester. Most recently, Aleidan et al [28] reported 3-times more frequent occurrence of subtherapeutic anti-Xa activity (C_max_: <0.6 U/mL) following enoxaparin in pregnancy (30%-36% frequency) than in nonpregnant state (6%-12% frequency), demonstrating that pregnancy is a statistically significant factor for C_max_/Dose reduction. These findings suggest that pregnancy-associated decrease in plasma antifactor Xa activity in those with APS unless the enoxaparin dose is increased to offset the anticipated C_max_ reduction. Reflecting a 30% to 50% expansion of apparent V [26,27], the mean residence time (a ratio between V and CL, proportional to elimination half-life) did not shorten [26]. Findings on dalteparin are similar. One study with a fixed prophylactic dose [29] showed a statistically significant reduction in C_max_, although statistical significance was not seen in the changes of apparent CL and AUC. The other study [25] for dalteparin described PK outcomes of a 3-fold dose escalation approach in pregnancy, showing increased anti-Xa activity in pregnancy by 50% to 70% when the dose increased. Probably due to large individual variations, increased CL did not reach statistical significance (Table 2). They did not report dose-adjusted values [25], but our estimates of AUC/Dose and C_max_/Dose were lower in pregnancy than nonpregnancy (Fig 2C,D). Although evidence is limited for both enoxaparin and dalteparin, there seems to be a sustained reduction of AUC/Dose throughout pregnancy and continuous reduction of C_max_/Dose as pregnancy progresses.

#### Chloroquine

A population PK study using plasma levels [20] showed a statistically significant increase in apparent CL of both chloroquine and its metabolite by about 30% during pregnancy, which led to significant AUC reduction by 25% to 45% (Table 3). Using whole blood-based data, Lee et al [30] did not observe statistically significant differences in AUC or C_max_. The other study by Chukwuani et al [32] contradicts these observations, showing inexplicably high DECQ plasma concentrations during pregnancy compared to nonpregnant state, although chloroquine disposition itself was similar to the other 2 studies (Table 3). Noteworthy is that these studies [20,30,31] included patients with malaria infection in malaria endemic areas, which indicates difficulties in extrapolating their results to nonmalaria settings. There is no report of chloroquine PK in pregnant women with APS.

#### Hydroxychloroquine

The only pregnancy PK study [32] was conducted using serum data in patients with lupus (Table 3). They showed pregnancy as a significant covariate positively affecting apparent CL with an estimated increase of as high as 60% in adherent pregnant women. As expected, their simulation results showed a decrease in serum HCQ concentrations by 30% to 40% throughout the pregnancy period [32]. In contrast, pregnancy did not significantly influence apparent V. Reflecting apparent CL increase and unchanged V, elimination half-life was estimated to be shortened by 10 days in pregnancy from 32 days in nonpregnant women. They also found that low serum concentrations of ≤100 ng/mL, strongly suggestive of nonadherence, were a significant determinant of preterm birth and associated with higher disease activity.

## DISCUSSION

Despite the diverse PK profiles, statistically significant PK changes of these 6 drugs during pregnancy are characterised by decrease in dose-standardised exposure levels: increases in apparent CL and/or significantly decreased AUC/Dose during pregnancy by as much as 50%. The findings inform drug management of APS during pregnancy, justifying dose increment approaches with monitoring of drug levels for some drugs such as heparins. We also note that postpartum recovery profiles of the altered PK parameters remain unclear, making it speculative to plan a dose de-escalation schedule.

To counteract pregnancy-associated CL increase and reduction of AUC, dose escalation or higher-dose regimens may be used to maintain target drug levels of those with a narrow therapeutic window, such as UFH and LMWH. Individualised dose increase in pregnancy is also suggested for LD-ASA [33]. However, evidence is limited, and searches for an optimal dosing strategy during pregnancy continue. For UFH and LMWH, thromboprophylaxis dosing rates during pregnancy are about 1.5- to 2-fold higher than nonpregnant state [34,35]. This dose escalation is consistent with the AUC reduction of UFH reported in a healthy volunteer study [24] (Table 2). However, above-threshold aPTT has been observed in 30% of women on thromboprophylaxis with the 2-fold dose escalation of UFH for prolonged antepartum admission, compared to 5% with a standard 5000 U/12h fixed dose [36]. Monitoring of coagulation status is clearly an indispensable element of thromboprophylaxis during pregnancy. At present, PK data on UFH for pregnant women remain extremely limited.

Defined from the rate of urinary excretion of a drug (ie, usually as an unchanged form), renal CL represents a propensity of the drug for kidney-mediated elimination via filtration plus net tubular secretion. Renal CL of LMWH ranges from 3% (dalteparin) to 15% to 20% (enoxaparin) of its total CL (reviewed by Frydman [37]), suggesting that renal excretion is a minor pathway. It is often interpreted that physiological renal hyper-perfusion during pregnancy is responsible for 50% increase in total CL of LMWH. However, if renal CL change is the sole mechanism for total CL increase, 3- to 10-fold increase of renal CL must be assumed, while the average increase of renal perfusion in pregnancy is only 50% (1.5-fold). This implies concomitant CL increase through nonexcretory routes such as the reticuloendothelial system including liver.

Reduction of enoxaparin C_max_/Dose and AUC/Dose during pregnancy is about 20% to 30% (Table 2) [[26], [27], [28]]. The limited data on dalteparin [25,29] are suggestive of similar changes. This implies that 1.5-fold higher doses per time are required to offset the reduction. Recently, results from a randomised controlled trial of LMWH in pregnant women with a history of venous thromboembolism have been reported, showing no prophylaxis superiority of intermediate doses (ie, 2-fold higher than a standard prophylactic dose) to a standard dose, although the intermediate dose may be more effective in the postpartum period [35]. However, a proportion of women with APS in study participants of the abovementioned trial [35] is not reported. In the absence of APS-specific data with large sample sizes, it seems reasonable to expect a 1.5-fold dose-increase requirement of LMWH thromboprophylaxis for pregnant women with APS to offset the exposure reduction. Although this is a fair inference, the clinical decision on dose increment certainly needs to be individualised depending on the treatment indications, risk factors, and patient's history, and moreover, monitoring of antifactor Xa levels is necessary. In addition, information on antifactor Xa levels at nonpregnant baseline and during pregnancy should help guide the antepartum - and postpartum care of these patients.

These drugs for APS do not show high plasma protein binding. Therefore, relative increases of the unbound fraction due to pregnancy-associated decrease of plasma protein concentrations are not large, which implies that the cause of increased CL is due to enhanced activity of drug elimination pathways. For aspirin, it is plasma cholinesterases [38]; for heparins, the reticuloendothelial system including sinusoidal endothelial cells of the liver, lymph nodes, and spleen through the scavenging receptor known as the hyaluronic acid receptor for endocytosis (stabilin-2) [39,40] in addition to renal tubular elimination as a minor route [41,42]; and for chloroquine and HCQ, liver enzymes [43] including CYP3A4 and CYP2C8 with contributions from GFR. Of these, pregnancy-associated activity increase was clearly documented for GFR, CYP3A4, and some other CYP enzymes (Note: reduced activity is reported for CYP1A1 and CYP2C19). Expansion of fluid volume in pregnancy leads to larger apparent V, lowering C_max_ to some extent, but V is independent of CL, exerting no influence on AUC.

After intestinal absorption, a fraction of aspirin in the presystemic circulation diffuses into platelets and acetylates platelet cyclooxygenase-1 (COX-1), causing irreversible inhibition [38,44]. In this process, aspirin is converted to salicylic acid. In parallel, aspirin in plasma is rapidly hydrolysed to salicylic acid via cholinesterase and tissue esterase, leading to its short plasma elimination half-life of <20 minutes. Relative to the rapid conversion of aspirin to salicylic acid, the metabolism of salicylic acid is slow with a longer half-life and becomes a rate-limiting step of aspirin–salicylic acid elimination, which makes it a convenient assay target. Its inhibition of COX-1 is reversible, and its concentrations after LD-ASA are low compared to high-dose anti-inflammatory therapy. Because the antiplatelet effect of LD-ASA relies mainly on aspirin-platelet contact in the presystemic circulation, interpretation of salicylic acid PK changes is difficult. The reported observations [22,23] suggest that plasma levels of both aspirin and salicylic acid become lower during pregnancy, which implies that CL of aspirin and salicylic acid is increased, aspirin F is decreased, or both. Whatever the mechanism, an aspirin pool in the presystemic circulation available for platelet uptake may be reduced in pregnancy, potentially lowering the therapeutic effects. A dose increase of LD-ASA therapy in pregnancy is suggested [23,33] but awaits further studies.

Like a prototype antimalarial chloroquine, HCQ shows extensive uptake into acidic lysosomal organelles of white blood cells and platelets [45]. Although its distribution into red blood cells (RBCs) is limited per cell, RBC-associated chloroquine and HCQ become also significant in whole blood-based assay due to the relative abundance of RBC counts per whole blood volume. Because of this, chloroquine and HCQ concentrations may become nearly 10-fold higher in whole blood than plasma [[45], [46], [47]]. Serum also shows higher concentrations than plasma due to the release of bound drugs into serum during platelet aggregation [45]. Consequently, CL and V derived from whole blood concentrations are only 1/4-1/10 of plasma-based CL and V. The matrix dependency of CL emerges because CL and V relate drug concentrations to drug elimination rate and amount in the body, respectively, which are independent of whatever matrix is chosen for concentration determination. Partly due to the complexity of the PK characteristics of chloroquine and HCQ, including a potential stereoselective immunomodulating activity [48], a target blood concentration for rheumatic conditions such as APS is not known. However, serum concentrations lower than 100 ng/mL of HCQ in pregnancy are suggestive of nonadherence, which may be associated with poor pregnancy outcomes [32].

Our study had characteristics that warrant discussion. To start, the database search strategy included 3 distinct terms: pregnancy, medications, and PKs. Because the review was identifying publications which included all 3 terms, some relevant publications may have been missed. To address this limitation, we tested with 2 terms, which led to the same results. Second, we excluded those with early-gestation control because pregnancy-associated PK changes often begin in the first trimester. Nonpregnancy PK data exist in the literature, but these PK reports are conducted mostly in adult males. The relative lack of PK and PD (pharmacodynamics) analyses in women remains a major challenge. Third, a meta-analysis of PK changes was not performed in our study due to variations in PK analysis methods and study design. Particularly, the selection of a nonpregnant non-APS control group poses a complex question as APS is an inflammatory autoimmune condition [[6], [7], [8]], which may have an impact on the activity of drug elimination pathways.

In summary, limited evidence suggests that these 6 drugs for APS undergo significant PK changes during pregnancy, which causes a reduction of drug exposure by as much as 2-fold with substantial variations. This may justify a dose escalation strategy with effect or surrogate monitoring, but information on clinical outcomes based on large enough sample sizes is scarce. In addition, details of the recovery profiles of the PK changes in the postpartum period are not well understood, making a de-escalation strategy speculative. Although clinical outcomes of the PK changes need to be elucidated, the lack of PK data in pregnancy and postpartum remains a significant challenge in women's health.

## CRediT authorship contribution statement

**Judith Zarek:** Writing – review & editing, Writing – original draft, Methodology, Investigation, Formal analysis, Data curation. **Avery Brydon:** Writing – original draft, Investigation, Data curation. **Karen Spitzer:** Writing – review & editing, Validation, Data curation. **Carl Laskin:** Writing – review & editing, Writing – original draft, Investigation, Conceptualization. **Shinya Ito:** Writing – review & editing, Writing – original draft, Visualization, Validation, Supervision, Resources, Project administration, Funding acquisition, Formal analysis, Data curation, Conceptualization.

## Competing interests

All authors have no known competing financial interests or personal relationships that could have appeared to influence the work reported in this paper.

## Appendix Supplementary materials

Image, application 1

## Footnotes

Supplementary material associated with this article can be found in the online version at doi:10.1016/j.ero.2025.03.001.
